# Why Aren't We Listening Yet? A Decade of Road Safety Begins Quietly

**DOI:** 10.1371/journal.pmed.1001464

**Published:** 2013-06-11

**Authors:** Tracey Pérez Koehlmoos

**Affiliations:** Headquarters, United States Marine Corps, Washington, District of Columbia, United States of America

## Abstract

Tracey Pérez Koehlmoos offers a personal view on road traffic crashes and the disappointing lack of visibility of the WHO-led Decade of Action for Road Safety.

*Please see later in the article for the Editors' Summary*

**Linked Essay**

This Perspective discusses the following Essay published in *PLOS Medicine*:

Redelmeier DA, McLellan BA (2013) Modern Medicine is Neglecting Road Traffic Crashes. PLoS Med 10(6): e10001463. doi:10.1371/journal.pmed.1001463

Tracey Pérez Koehlmoos offers a personal view on road traffic crashes and the disappointing lack of visibility of the WHO-led Decade of Action for Road Safety.

The dogs bark, the bell rings, I shuffle to the door in my pajamas to discover a Jakarta police officer who stammers, “Madam, hospital.” Fifteen minutes later, the drawer slides open. Yes, that is my husband on the morgue tray. I touch his face; he is still warm. ‘Get up,’ I think. I kiss his forehead. ‘Get up!’ I want to shout. Larger than life, a force of nature — how can a great man fit in such a small space? Every plan, every hope, and every dream for the rest of my life died with him in the instant our car crumpled like an accordion, and so I become a widow of the road.

This tragedy of road safety, so often a peripheral issue, became my issue. Over the last 18 months since my husband died, I have learned that I am not alone. Tragedy has made road safety an issue for many others—Mothers Against Drunk Driving (MADD), the Association for Safe International Road Travel, and individuals who have yet to find a voice amidst their lingering grief. I am a researcher and so I have looked into who is doing what, what is the progress, what are the goals, and what I can do to assist.

Each year, approximately 1.3 million people worldwide die due to road traffic crashes, most of them between 15 and 44 years of age; an additional 20 to 50 million people are injured or disabled [1]. By 2030, road traffic injuries are predicted to be the fifth leading cause of death globally. Further, there is a development disparity in that low- and middle-income countries account for 50% of the world's motor vehicles but more than 90% of the mortality burden for road traffic crashes [1]–[3]. In addition to the deaths among all car and motorcycle drivers and passengers, 27% of all road traffic deaths occur in pedestrians or bicyclists; in some low- and middle-income countries, this number spikes to more than 75% of deaths [4]. Adding to the economic disparity is the slip by many families into poverty through the loss of a bread winner, prolonged medical expenditures, or long-term care of a disabled loved one as a result of road traffic crashes [7].

We should be alarmed by Redelmeier and McLellan's article in this week's *PLOS Medicine* that argues modern medicine is neglecting road traffic crashes [6]. The issue of road safety is enormous—so enormous that perhaps it is easily forgotten as each among us hops into our car to drive to work, crosses the street to go to the store, or presses on with our daily life, unaware of the risk. Only 28 countries, accounting for just 7% of the world's population, have comprehensive road safety laws on five key risk factors: drinking and driving; speeding; and failing to use motorcycle helmets, seat-belts, and child restraints [4]. There is a cache of proven, cost-effective interventions, such as speed bumps and lowering speed limits, but the pervasive lack of evidence from developing countries hinders the implementation of these strategies where they are most needed [8].

It is the Decade of Action for Road Safety (2011–2020). Who knew? It has arrived so quietly perhaps only those of us who have experienced a death on the roads are in tune with this effort. How much better for the target audience to be everyone who lives where there are roads, not just vehicle owners. Perhaps our focus on the rapidly approaching deadline for the ambitious Millennium Development Goals causes us to overlook the risk in our daily lives. In March 2013, The World Health Organization released a global status report on road safety. Each year to 2020, WHO will release a road traffic report with a different area of focus [4]. While the exact targets of the decade are hard to pin down, other than a vague ‘stabilize and then reduce global road traffic fatalities by 2020’, the plan is to reduce deaths on the road through better road safety management, placing emphasis on safer roads and mobility, safer vehicles and safer road users, and improved post-crash reporting [5].

Road traffic safety is the quintessential issue that demands a whole of government/whole of society approach. Although this issue demands attention at the highest international level, no international organisation currently looks solely at the complex issue of road traffic safety (within WHO, this important issue is included in the Violence and Injury Prevention program). Although road traffic crashes fall outside of the scope of health systems, road traffic injuries have an enormous impact on health systems. The impact is felt particularly in low-income countries where systems of traffic law enforcement, urban planning, and justice may be less developed and where more of the population is vulnerable to injury because people are more likely to walk or to ride in substandard vehicles (bicycles, rickshaws, motorcycles, three wheelers). With all of the public health attention focused on ‘Wear a Condom’ and ‘Get a Mammogram’, we should perhaps spend more time focusing on ‘Drive Carefully, ‘ ‘Look both ways before you cross,’ and ‘Wear your seat belt.’

Each among us must raise the precedence of road traffic safety to our policy makers, program officials, media contacts, and development partners. Road Safety Week was 6–12 May 2013. This year is dedicated to pedestrian safety. How can we work to ensure the safety of pedestrians? We can start at home by driving more attentively in areas that are crowded or where children might be playing unattended. Commit to not talking on mobile phones or texting while driving. Lobby for the creation of pedestrian bridges and enforcement of cross-walk safety standards or for the installation of speed bumps. We must raise our voices so the decade of road crashes is heard loud and clear.
